# 2D Tsallis Entropy for Image Segmentation Based on Modified Chaotic Bat Algorithm

**DOI:** 10.3390/e20040239

**Published:** 2018-03-30

**Authors:** Zhiwei Ye, Juan Yang, Mingwei Wang, Xinlu Zong, Lingyu Yan, Wei Liu

**Affiliations:** 1 School of Computer Science, Hubei University of Technology, Wuhan 430068, China; 2 School of Remote Sensing and Information Engineering, Wuhan University, Wuhan 430079, China

**Keywords:** 2D Tsallis entropy, image segmentation, Bat algorithm, chaotic process, Levy flight

## Abstract

Image segmentation is a significant step in image analysis and computer vision. Many entropy based approaches have been presented in this topic; among them, Tsallis entropy is one of the best performing methods. However, 1D Tsallis entropy does not consider make use of the spatial correlation information within the neighborhood results might be ruined by noise. Therefore, 2D Tsallis entropy is proposed to solve the problem, and results are compared with 1D Fisher, 1D maximum entropy, 1D cross entropy, 1D Tsallis entropy, fuzzy entropy, 2D Fisher, 2D maximum entropy and 2D cross entropy. On the other hand, due to the existence of huge computational costs, meta-heuristics algorithms like genetic algorithm (GA), particle swarm optimization (PSO), ant colony optimization algorithm (ACO) and differential evolution algorithm (DE) are used to accelerate the 2D Tsallis entropy thresholding method. In this paper, considering 2D Tsallis entropy as a constrained optimization problem, the optimal thresholds are acquired by maximizing the objective function using a modified chaotic Bat algorithm (MCBA). The proposed algorithm has been tested on some actual and infrared images. The results are compared with that of PSO, GA, ACO and DE and demonstrate that the proposed method outperforms other approaches involved in the paper, which is a feasible and effective option for image segmentation.

## 1. Introduction

Image analysis and computer vision are core topics in artificial intelligence, which have been deeply studied and obtained a wealth of achievements [1]. Image segmentation is a primary task in image analysis and computer vision. It is a process that partitions the whole image into several regions based on compatibility and dissimilarity existing between the pixels of the input image [2,3]. Thresholding is a commonly used method for carrying out image segmentation based on the different intensities in the foreground and background regions of an image [4,5]. A variety of thresholding methods have been proposed to perform the task of image segmentation [6,7]. Galland et al. [8] utilized Fisher probability density functions for image segmentation. Wei et al. [9] used a novel adaptive thresholding method based on multi-directional gray-scale wave transformation for image segmentation. The image could be regarded as a digital signal with certain probability distribution; as a result, many entropy based thresholding approaches have been put forward, which seek the thresholds that separate the gray-level regions of an image in an optimal manner on the basis of some discriminating criteria such as maximum entropy and cross entropy. Li and Tam [10] presented a minimum cross-entropy thresholding method to obtain the binary image as indicative of preservation of information. Sahoo et al. [11] presented a general technique for thresholding of digital images based on Renyi’s entropy. Tsallis entropy is called non-extensive entropy, which has been studied for a possible extension of Shannon’s entropy to information theory [12,13]. This study has brought a similarity between the Shannon’s entropy and Boltzmann/Gibbs entropy functions [14]. Khader et al. [15] used a multicomponent Jensen–Tsallis similarity measure for nonrigid registration of diffusion tensor images, which had a better performance than the affine registration method based on mutual information. In addition, it is also pointed out in [16] that Jensen–Tsallis divergence is generalizable and symmetric for any optional number of datasets or probability distributions and it has the feasibility of assigning weights to the distributions.

However, entropy-based methods based on 1D histograms do not utilize the spatial correlation information within the neighborhood. When the proportion of the target area is very small, its threshold of the image is very easy to drift or shift. In addition, the segmented result is also easily influenced by noise. Consequently, a group of entropy-based methods based on 2D histograms are proposed to solve the problem. Brink [17] presented an approach that evaluates 2D entropy based on the two-dimensional (local average grey-scale) histogram or scatterplot. Sahoo and Arora [18] presented a common thresholding method based on 2D Renyis entropy. Wu et al. [19] proposed a modified 2D entropy thresholding method, and its fast iterative algorithm.

All of the above 2D entropy thresholding methods can obtain better segmented results than the 1D entropy thresholding method. However, as the number of dimension increases, the computational complexity greatly increases as the number of thresholds increases [20]. Obviously, the alternate solution is to employ computational techniques [21]. Evolutionary computation is a subfield of artificial intelligence that involves continuous optimization and combinatorial optimization problems, which has been previously used to perform 2D entropy-based image segmentation. Abdel-Khalek et al. [22] presented a 2D Tsallis and Renyi entropies method of image segmentation based on genetic algorithm (GA). Zhou et al. [23] proposed a two-dimensional Otsu image segmentation based on a modified firefly algorithm. Soham Sarkar [24] presented a two-dimensional histogram and maximum Tsallis entropy based on differential evolution algorithm (DE) to improve the separation between objects. Jiang et al. [25] suggested combining artificial fish-swarm algorithm (AFSA) and two-dimensional Fisher evaluation function to improve image the threshold segmentation algorithm. Similar to other 2D entropy-based approaches, 2D Tsallis entropy has been used for image segmentation [13], whose efficiency still needs to be improved.

The Bat algorithm (BA) is a population-based random search algorithm that imitates the intelligent behavior of organisms to solve complex problems. Yang has testified its performance on some benchmark functions [26]. However, since its local search scope is too narrow; the convergence speed of the primary BA is slow, which might be improved by some strategies.

Chaos, defined as highly unreliable motion in limited phase space, often appears in deterministic nonlinear dynamic systems, the motion of which is similar to stochastic processes [27]. Taking advantage of its easy implementation and extensive local search scope, any variable in the chaotic space can travel periodically over the whole space of interest. Feng et al. [28] presented the application of appropriate chaotic map and Gaussian perturbation can significantly improve the overall performance and the solution quality of the algorithm. Nowadays, the chaotic process has been widely used in evolutionary computational techniques [29,30]. Adarsh et al. [31] proposed solving the economic dispatch problem involving a number of equality and inequality constraints such as prohibited operating zones and power balance. Suresh [32] proposed a modified variant of Darwinian particle swarm optimization algorithm based on Chaotic functions. Wang [33] proposed a band selection method based on chaotic binary coded gravitational search algorithm to reduce the dimensionality of airborne hyperspectral images. Mlakar et al. [34] proved that the chaotic maps method in differential evolution (DE) applied to gray-level image thresholding prevails over the traditional randomized method. Wang et al. [35] proposed the novel chaotic cuckoo search optimization, which has turned out to be a meta-heuristic algorithm with comparable and superior performance. Wang et al. [36] improved the solution quality and convergence speed using chaotic maps in the cuckoo search (CS) algorithm. Zhang et al. [37] proposed a hybrid chaotic ant swarm algorithm, which performs better in the application of the heat exchanger networks synthesis. Oliva et al. [38] proposed the chaotic whale optimization algorithm for the parameter estimation of photovoltaic cells, which proves that chaotic maps can enhance computing ability and mechanically adapt the internal parameters of the optimization algorithm. Koupaei et al. [39] proposed a new algorithm to solve nonlinear optimization problems by combining chaotic mapping capability and a gold segmentation search method that has been proved to be an effective and efficient optimization algorithm.

Levy flight is a way to forage for random walks existing among animals, the walking steps of which satisfied a heavy tail distribution. It is also an ideal foraging search for exploring short distances and occasionally long walks. Therefore, it is widely utilized to improve the optimization algorithm [40]. Yan et al. [41] developed the particle swarm optimization (PSO) algorithm by combining the characteristics of random learning mechanism and Levy flight. Jensi et al. [42] improved PSO with Levy flight for the global optimization. Heidari [43] modified the grey wolf optimizer with Levy flight for optimization tasks.

In this paper, a modified chaotic Bat algorithm (MCBA) is used to perform the 2D Tsallis entropy based method for image segmentation. In addition, the performance of the proposed method is compared with some previously presented 1D and 2D histogram based image segmentation methods, and a traditional Bat algorithm based 2D Tsallis entropy method. The experimental results display that the proposed method outperforms the other approaches from the subjective and objective viewpoints.

The remainder of the paper will be structured as below: Section 2 introduces image thresholding based on Tsallis entropy. Section 3 describes the modified chaotic Bat Algorithm in brief. Section 4 presents the fundamental viewpoint of MCBA-based 2D Tsallis entropy thresholding method. In Section 5, simulation results and discussion are displayed. Finally, conclusions are drawn in Section 6.

## 2. Image Thresholding Based on Tsallis Entropy

### 2.1. Definition of 1D Tsallis Entropy

In information theory, entropy is the measurement of the indeterminacy in a random variable [44]. The term mostly refers to the Shannon entropy in this case. Shannon entropy quantifies the expected value of the information that is comprised in a message, and it is the average unpredictability in a random variable equal to its information content. The most commonly used Shannon entropy was defined as Equation (1):

(1) $$S_{Shannon}=-\sum_{i=1}^{n}p_{i}\ln p_{i}.$$

By following the multi-fractal concepts, Tsallis entropy is an extension of the standard Boltzmann/Gibbs entropy. In 1988, Constantino Tsallis utilized it as a support for generalizing the standard statistical mechanics [45], which could be applied to a non-extensive system in view of a general entropic formula as Equation (2):

(2) $$S_{q}=\frac{1-\sum_{i=1}^{n}{\left(p_{i}\right)}^{q}}{q-1},$$

where *n* indicates total times of the system and *q* is the measure of degree of non-extensivity of the system called Tsallis parameter or even entropic index. Different values of the parameter q have an effect on the image segmentation result [46]. When the whole system consists of two independent subsystems *A* and *B*, this entropic form can be extended for a statistical independent system by a pseudo-additive entropic rule as Equation (3):

(3) $$S_{q}\left(A+B\right)=S_{q}\left(A\right)+S_{q}\left(B\right)+\left(1-q\right)S_{q}\left(A\right)S_{q}\left(B\right).$$

### 2.2. 2D Histogram

Assume that the size of a digital image is M×N, and f(x,y) indicates the gray value of the pixel located at the point (x,y). g(x,y) represents the average gray value of the pixel located at the point (x,y) in the adjacent field of k×k (*k* is usually set as 3) [47]. The average gray value for the k×k neighborhood of each pixel is calculated as Equation (4):

(4) $$g\left(x,y\right)=\frac{1}{k^{2}}\sum_{m=-\frac{k}{2}}^{\frac{k}{2}}\sum_{n=-\frac{k}{2}}^{\frac{k}{2}}f(x+m,y+n).$$

The pixel’ s gray value f(x,y), and the average of its neighborhood g(x,y), are utilized to construct a 2D histogram as Equation (5):

(5) $$p\left(i,j\right)=\frac{1}{M\times N}\left\{n_{ij}|f\left(x,y\right)=i,g\left(x,y\right)=j;i,j\in \left(0,L-1\right)\right\},$$

where M×N indicates the size of test image, $n_{ij}$ represents the pixel number of which the gray value is *i*, and the average gray value in the neighborhood is *j*. p(i,j) indicates the 2D histogram function of test image. *L* is the maximum gray values of the test images. In our test images, *L* is 255.

**Figure 1:**
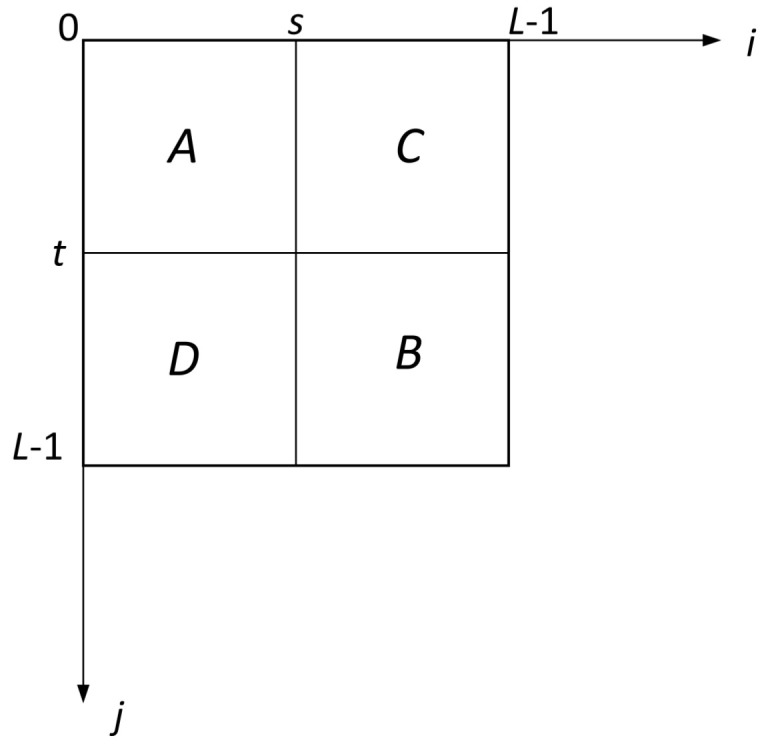
The 2D histogram plane.

The threshold is obtained through a vector (t,s), where *t* indicates the threshold of the gray level and *s* indicates the threshold of the average gray level in the neighborhood. Using the 2D histogram function $P_{ij}$, a surface can be described that will have two peaks and one valley. The object and background correspond to the peaks and can be divided by selecting the vector (t,s) that maximizes a appropriate standard function. Using this vector (t,s), the domain of the histogram is separated into four quadrants, shown in Figure 1.

It is indicated that the area *A* by [0,t]×[0,s], the area *B* by [t+1255]×[s+1255], the area *C* by [0,t]×[s+1255], and the area *D* by [t+1255]×[0,s]. Since two areas, *C* and *D*, contain noise and inessential information, they are ignored in the calculation. Owing to the areas *A* and *B* containing the object and the background, they are independently distributed, normalizing their probability values in each case so that the total probability of each region is 1. The normalization is completed by utilizing a posteriori class probabilities, $P_{A}\left(t,s\right)$ and $P_{B}\left(t,s\right)$ are defined as Equation (6):

(6) $$P_{A}\left(t,s\right)=\sum_{i=0}^{t}\sum_{j=0}^{s}p(i,j)\;P_{B}\left(t,s\right)=\sum_{i=t+1}^{255}\sum_{j=s+1}^{255}p(i,j),$$

where the contribution of the areas that comprises the edges and noise information can be ignored; hence, $P_{A}\left(t,s\right)$ is approximately equal to $P_{B}\left(t,s\right)\approx 1-P_{A}\left(t,s\right)$.

### 2.3. Definition of 2D Tsallis Entropy

Assume that the test image can be segmented as two independent parts: object sets *O* and background sets *B*, the 2D Tsallis entropy interrelated with object and background sets are defined by Equations (7) and (8):

(7) $$S_{q}\left(O\right)=\frac{1-\sum_{i=0}^{t}\sum_{j=0}^{s}{\left(\frac{p(i,j)}{P_{A}\left(t,s\right)}\right)}^{q}}{q-1},$$

(8) $$S_{q}\left(B\right)=\frac{1-\sum_{i=t+1}^{255}\sum_{j=s+1}^{255}{\left(\frac{p(i,j)}{P_{B}\left(t,s\right)}\right)}^{q}}{q-1}.$$

Consequently, the definition of 2D Tsallis entropy of the whole image is as Equation (9):

(9) $$S_{q}\left(t,s\right)=S_{q}\left(O\right)+S_{q}\left(B\right)+\left(1-q\right)S_{q}\left(O\right)S_{q}\left(B\right).$$

Three different entropies can be defined by different values of *q*. For q<1, the Tsallis entropy becomes a subextensive entropy, where $S_{q}\left(O+B\right)<S_{q}\left(O\right)+S_{q}\left(B\right)$; for q=1, the Tsallis entropy reduces to an standard extensive entropy, where $S_{q}\left(O+B\right)=S_{q}\left(O\right)+S_{q}\left(B\right)$; for q>1, the Tsallis entropy becomes a superextensive entropy, where $S_{q}\left(O+B\right)>S_{q}\left(O\right)+S_{q}\left(B\right)$ [48]. The paper mainly focused on enhancing efficiency of 2D Tsallis entropy thresholding; therefore, *q* is set as a constant value, that is q=2.

In order to obtain the optimal segmentation threshold $\left(t^{*},s^{*}\right)$, the objective function is defined to maximum the above criterion function $S_{q}\left(t,s\right)$ as Equation (10):

(10) $$\left(t^{*},s^{*}\right)=Arg\;\max S_{q}\left(t,s\right),\;t,s\in \left(0,255\right).$$

## 3. The Modified Chaotic Bat Algorithm

Nowadays, meta-heuristic algorithms are becoming an efficient way for solving tough optimization problems [49]. For the most part, meta-heuristic algorithms make few or no assumptions about the optimization problem to be solved and are able to make an extensive search in candidate spaces. Behaviors of physical systems or biological systems in nature have bred most heuristic and meta-heuristic algorithms, such as ant colony optimization algorithm (ACO) algorithm, PSO and simulated annealing. In 2010, Xin-She Yang developed a novel bat inspired meta-heuristic optimization algorithm [50]. Owing to the initialization process being random and the generated sequences not being distributed uniformly, the experimental results on the benchmark function show that the basic Bat algorithm can easily fall into local optimum [31]. The chaotic Bat algorithm (CBA) is a very efficient method for solving the optimization problem. However, for the evolutionary algorithm, due to the chaotic process, is usually taken after each iteration is completed. In the traditional Bat algorithm, a random walk is used to make a local search, and the chaotic process largely extends the search space of the whole algorithm. The previous local search will be out of action, and the convergence rate of the algorithm is also significantly reduced. On the other hand, the local search ability is mainly influenced by the parameter ε; ε is a manually set parameter in the initialization step and cannot be changed during the whole iterative processes. ε that is too big or too small will all lead to a case that the algorithm can not obtain the optimal solution. Levy flight was proposed as a local search strategy by Yang and Deb in 2009 [51]. They found that the random walk style search performed better through Levy flights rather than a simple random walk, which is more efficient in searching the solve space, and its step length is much longer in the long run. More importantly, Levy flight does not need to set any parameters. All of these advantages make Levy flight have better local search ability.

### 3.1. Overview of the Basic Bat Algorithm

The Bat algorithm (BA) is inspired by ultrasonic detection behavior of bats, in which virtual bats are deemed as search agents to find the optimal solution for practical problems. Simulating bats to detect prey and avoid obstacles, the basic mathematical model of the Bat algorithm could be defined as follows:

Assume that virtual bats fly randomly with velocity $V_{i}$ at position $X_{i}$ and go hunting with a settled frequency of $f_{\min}$, variable wavelength λ and loudness $A_{0}$. The bats are able to automatically alter the wavelength and the pulse emission rate r∈[0,1] on the basis of the distance from the targets. The loudness varies from a maximum value $A_{0}$ to a minimum value $A_{\min}$, which both are constants. In addition, $f\in [0,f_{\max}]$ and the plus rate ranges in Bat algorithm is between 0 and 1, where 1 means the maximum rate of pulse emission and 0 stands for no pulse.

#### 3.1.1. Movement of Virtual Bats

Rules that update new solutions $X_{{}_{i}}^{t}$ and velocities $V_{{}_{i}}^{t}$ at time step *t* in a multi-dimensioned search space are defined as Equations (11)–(13) [52,53]:

(11) $$f_{i}=f_{\min}+\left(f_{\max}-f_{\min}\right)\beta ,$$

(12) $$V_{{}_{i}}^{t}=V_{{}_{i}}^{t-1}+\left(X_{{}_{i}}^{t}-X^{*}\right)f_{i},$$

(13) $$X_{{}_{i}}^{t}=X_{i}^{t-1}+V_{i}^{t},$$

where β is a random vector in [0,1] and $x_{i}^{t}$ and $v_{i}^{t}$ are on behalf of the new solutions and the velocities value in time step *t*, respectively. In Equation (12), $X^{*}$ denotes the current global optimal solution in all solutions engendered by *n* bats. Here, parameter $f_{i}$ is utilized to adjust the velocity change while fixing the wavelength $\lambda_{i}$, depending on the form of the problem of interest, which is randomly generated in the range of $\left[f_{\min},f_{\max}\right]$.

For the sake of fine search, the local search operation of the Bat algorithm has been designed as Equation (14):

(14) $$X_{new}=X_{old}+\varepsilon A^{t},$$

where ε∈[0,1] is a random number, and the average loudness of all bats are represented by $A_{t}=<A_{i}^{t}>$ at time step *t*.

#### 3.1.2. Loudness and Pulse Emission

As the algorithm is executed, loudness $A_{i}$ and rate $r_{i}$ requires to be renewed according to the time step *t*. In the hunting process, once the prey is captured by the bat, $A_{i}$ will get decreased while $r_{i}$ will get increased. Usually, $A_{i}$ might be fit with any effective value:

(15) $$A_{i}^{t+1}=\alpha A_{i}^{t},\begin{array}{cc} & r_{i}^{t+1}=r_{i}^{0} \end{array}\left[1-\exp\left(-\gamma t\right)\right].$$

In Equation (15), α and γ are usually set as constants. For any α and γ in the range of 0<α<1 and γ>0, we have Equation (16) as below:

(16) $$A_{i}^{t}\to 0,\;r_{i}^{t}\to r_{i}^{0},\;as\;t\to \infty .$$

Generally, α is set equal to γ. Parameters needs to be fine-tuned from experiments.

### 3.2. Chaotic Process

Chaos is an irregular nonlinear phenomenon in nature, which is defined as the highly unstable unpredictable motion of deterministic systems in restricted phase space. Yang et al. [53] proposed chaotic optimization algorithms for global optimization that utilized chaotic variables instead of random variables. In these algorithms, due to the non-repetition and ergodicity of chaos, they can achieve an overall search at a higher speed than probabilistic random searches. Accordingly, a nonlinear system is said to be chaotic if it exhibits sensitive dependence on initial conditions, which is generally exhibited by systems containing multiple elements with nonlinear interactions, and has an infinite number of different periodic responses [54]. Additionally, complex systems as well as some logistic equations are utilized to generate chaotic sets. In addition, some of the well-known chaotic maps, as illustrated from Equations (17) to (27), are as below:

1. Logistic map:
  (17) $$x_{n+1}=\mu x_{n}\left(1-x_{n}\right),$$
  where $x_{n}$ denotes the value of the chaotic variable *x* at the *n*-th iteration, x∈(0,1) , and μ is the bifurcation parameter, μ∈(0,4].
2. Sine map:
  (18) $$x_{n+1}=\frac{\mu }{4}\sin\left(\pi x_{n}\right),$$
  where $x_{n}$ denotes the value of the chaotic variable *x* at the *n*-th iteration, x∈(0,1) , and μ is the bifurcation parameter, μ∈(0,4].
3. Sinusoidual map:
  (19) $$x_{n+1}=\mu x_{n}^{2}\sin\left(\pi x_{n}\right),$$
  where $x_{n}$ denotes the value of the chaotic variable *x* at the *n*-th iteration, x∈(0,1) , and μ is the bifurcation parameter, μ∈(0,2.5].
4. Singer map:
  (20) $$x_{n+1}=\mu \left(7.86x_{n}-23.31x_{n}^{2}+28.75x_{n}^{3}-13.3x_{n}^{4}\right),$$
  where $x_{n}$ denotes the value of the chaotic variable *x* at the *n*-th iteration, x∈(0,1) , and μ is the bifurcation parameter, μ∈[0.9,1.08].
5. Sinus map:
  (21) $$x_{n+1}=2.3{\left(x_{n}\right)}^{2\sin(\pi x_{n})},$$
  where $x_{n}$ denotes the value of the chaotic variable *x* at the *n*-th iteration, x∈(0,1).
6. Chebyshev map:
  (22) $$x_{n+1}=\left(\cos\left(k\cos^{-1}\left(x_{n}\right)\right)+1\right)/2,$$
  where $x_{n}$ denotes the value of the chaotic variable *x* at the *n*-th iteration, x∈(0,1) , and *k* is the degree parameter, k∈(0,4].
7. Circle map:
  (23) $$x_{n+1}=\left(x_{n}+\alpha -\left(\frac{\beta }{2\pi }\right)\sin\left(2\pi x_{n}\right)\right)\;\mathrm{mod}\;1,$$
  where $x_{n}$ denotes the value of the chaotic variable *x* at the *n*-th iteration, x∈(0,1) , while α and β are the control parameters; here, α=0.5, β=0.2.
8. Dyadic map:
  (24) $$x_{n+1}=\left(2x_{n}\right)\;\mathrm{mod}\;1,$$
  where $x_{n}$ denotes the value of the chaotic variable *x* at the *n*-th iteration, x∈(0,1) .
9. Gaussian map:
  (25) $$x_{n+1}=\left\{\begin{array}{cc} 0, & x_{n}=0,\\ \left(\frac{\mu }{x_{n}}\right)\;\mathrm{mod}\;1, & x_{n}\neq 0, \end{array}\right.$$
  where $x_{n}$ denotes the value of the chaotic variable *x* at the *n*-th iteration, x∈(0,1), and μ is the bifurcation parameter, which can be set as arbitrary constant.
10. Iterative map:
  (26) $$x_{n+1}=\left(\sin\left(\frac{\mu \pi }{x_{n}}\right)+1\right)/2,$$
  where $x_{n}$ denotes the value of the chaotic variable *x* at the *n*-th iteration, x∈(0,1), and μ is the bifurcation parameter, μ∈(0,1].
11. Tent map:
  (27) $$x_{n+1}=\left\{\begin{array}{cc} \mu x_{n}, & x_{n}<0.5,\\ \mu \left(1-x_{n}\right), & x_{n}\geq 0.5, \end{array}\right.$$
  where $x_{n}$ denotes the value of the chaotic variable *x* at the *n*-th iteration, x∈(0,1), and μ is the bifurcation parameter, μ∈(0,2].

### 3.3. Levy Flight

Levy flight is a Markov chain process, which is different from regular Brownian motion that the lengths of individual jumps are distributed with the probability density function. Various research indicates that the flight behaviour of many organisms has demonstrated the typical characteristics of Levy flights [55]. Rhee et al. [56] has shown that fruit flies explore their landscape through a series of straight flight paths punctuated by a abrupt $90^{\circ }$ turn, which leads to a Levy-flight-style intermittent scale free search pattern. To generate a new solution $x^{\left(t+1\right)}$, Levy flight can be defined as Equation (28):

(28) $${x_{i}}^{\left(t+1\right)}={x_{i}}^{\left(t\right)}+\alpha ⊕Levy\left(\lambda \right),$$

where $x_{i}^{t+1}$ denotes the new solution relying on the current location and the transition probability. The product ⊕ means entry-wise multiplications. α is the step size that needs to be connected with the search space—in most cases, α>0, for controlling the step size. This entry-wise product is similar to those employed in PSO, and the random walk by Levy flight is more efficient to explore the searching space, and it has a much longer step length in the long term.

In essence, Levy flight provides a random walk while the random step length is drawn from a Levy distribution, which has an infinite variance with an infinite mean as Equation (29):

(29) $$Levy∼u=t^{-\lambda }\;\;\;\;\;\left(1<\lambda \leq 3\right).$$

In Equation (29), the consecutive steps of a bat are from a random walk process that obeys a power law step-length distribution with a heavy tail. Some of the new solutions should be produced via Levy flight round the optimal solution acquired by far; this will speed up the local search.

The main idea of MCBA is that, after updating the velocity vector $V_{i}$ and position vector $X_{i}$, the chaotic process is utilized to make a random search for each bats, and, finally, the local search strategy by using Levy flight is adopted for the best solutions in the current generation to quickly obtain the optimal solution.

## 4. The Proposed Methodology

In this paper, a modified chaotic Bat algorithm (MCBA) is proposed to complete the task of image segmentation. MCBA is employed to search the optimal threshold values, which is used for image segmentation. Here, the algorithm is utilized to search the best thresholding pair through maximizing the 2D Tsallis entropy. The initialization of the bat population is generated with *n* number of solutions, each of which is a D-dimension vector. For each solution representing 2D candidate threshold, D is set to 2. $X_{i}$ denotes the *i*-th bat position in the population, which indicates a candidate threshold pair and its fitness will be measured by 2D Tsallis function. The basic procedures of image segmentation by using MCBA-based 2D Tsallis entropy algorithm can be depicted as follows:

Input:   The set of generated position vectors $X_{i}\left(i=1,2,\ldots ,n\right)$ as the parameters *t*, *s*.
Output: The final segmented image with selected thresholds based on the optimal parameters.

Step 1: Initialization
  Set the control parameter value. Initialize velocity vectors $V_{i}$, pulse rates $r_{i}$ and the loudness $A_{i}$. Define pulse frequency $f_{i}$ at $X_{i}$, iterations of MCBA k=0.
Step 2: Calculate the fitness value
  Compute the value of objective function $S_{q}\left(t,s\right)$ using Equation (9).
Step 3: Reproduction loop
  Reproduction loop: k=k+1
Step 4: Movement of virtual bats
  4.1: Generate new solutions by adjusting frequency, and update velocities and locations/solutions by using Equations (11)–(13).
  4.2: Make a chaotic process by using one of Equations (17)–(27).
  4.3: Compute the value of objective function $S_{q}\left(t,s\right)$ using Equation (9).
  4.4: Make a local search by Levy flight using Equation (28).
Step 5: Loudness and pulse emission
  If $A_{i}>1.4\;\&\&\;H\left(X_{i}\right)<H\left(X^{*}\right)$
    Accept the new solutions.
  Increase $r_{i}$ and reduce $A_{i}$ by using Equations (15) and (16).
Step 6: Update the current best parameters
  Rank the bats and find the current best $X^{*}$.
Step 7: End of the algorithm
  If (*k* < Max_gen)
    go to Step 3.
  Else
    output the segmented image based on the optimal parameters.

According to the operational process of meta-heuristic algorithm, the computational results of the Bat algorithm depend on parameters setting in some extent; fine tuning of the parameters setting can produce a better result. Among them, Table 1 show the parameters used in the Bat algorithm.

## 5. Simulation Results and Discussion

In order to evaluate the performance of the algorithms on the actual images accurately and effectively, the proposed algorithm and some classical optimization algorithms will be tested on a set of images. In the first part, some natural images are utilized for testing the mentioned algorithms, the objects, foreground and background of which can be accurately separated by suitable thresholds. In addition, some infrared target images were selected in this paper, which are obtained by measuring the heat radiated from the object, which can resolve the consistency judgment of the same target and distinguish the objects by gray levels.

In this section, for the sake of showing the advantages of the proposed method, five nature images respectively named “AIRPORT”, “PLANE”, “BIRD”, “SKIMAN”, “MRI” and five infrared images respectively named “SHIP”, “FIELD”, “TARGET”, “PISTOL”, “PERSON” are used to evaluate the segmentation technique based on the MCBA-based 2D Tsallis entropy algorithm. We present some contrastive experimental results, including illustrative examples and performance evaluating tables, which clearly demonstrate the advantages of the proposed method. To make purposeful yet effective comparison for the optimization ability, the results of MCBA are respectively compared with GA, PSO, ACO, DE, and BA. All the test images are classified as two different categories; all the algorithms are evaluated by using Equation (10) as the objective function. Table 2, Table 3, Table 4 and Table 5 show the parameters setting of GA, PSO, ACO and DE, and results are put in Table 6, which include average and variance of the fitness value by 50 independent runs for each algorithm. The MATLAB code to generate these thresholds is available as Supplementary Material.

Furthermore, to make purposeful yet effective comparison for the segmentation ability, results of the proposed method are respectively compared with 1D Fisher, 1D maximum entropy, 1D cross entropy, 1D Tsallis entropy, fuzzy entropy, 2D Fisher, 2D maximum entropy and 2D cross entropy. All the original images and segmented results are shown in Figures 4–13.

In order to evaluate the segmentation objectively, the absolute error ratio [57] is used as the performance indicator in this paper, which is defined as follows:

(30) $$r_{err}=\frac{n_{diff}}{N}\times 100\%.$$

The absolute error $n_{diff}$ is defined as the absolute difference of the number of target pixels between the optimum thresholding image and the thresholding image obtained by each methods. The optimum threshold image and the corresponding threshold are acquired manually through visual inspection.

### 5.1. Comparison of Optimization Ability

For testing the effectiveness of the proposed MCBA based 2D Tsallis entropy algorithm, five nature images and five infrared images have been implemented separately for this method. The evaluation metrics for examining the performance of the method are made with the choice of the absolute error ratio, which is utilized to ensure the quality of the thresholding images. In order to evaluate and compare its optimization ability, MCBA is compared with other evaluation algorithms such as GA, PSO, ACO, DE, BA and CBA.

**Figure 2:**
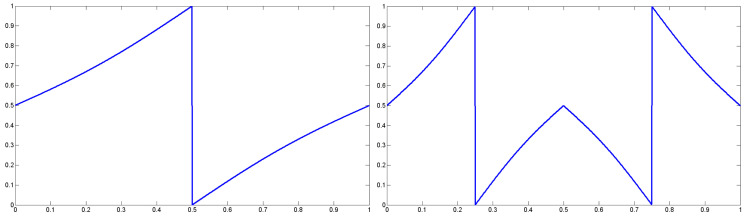
Mapping curve of the basic circle map and the piecewise circle map.

**Figure 3:**
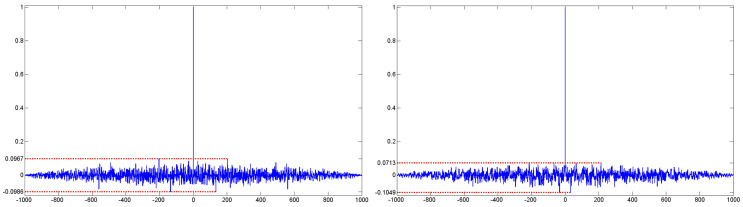
Autocorrelation curve of the basic circle map and the piecewise circle map.

Table 6 shows the average fitness value of 2D Tsallis entropy respectively using GA, PS, ACO, DE, BA for 10 test images. The fitness value of GA, PSO and ACO is distinctly worse than that of DE and BA, and the maximum difference of the average fitness value between them is more than 2.9. For “MRI” and “GUN” images, the fitness value of DE and BA is very similar. However, for all of the 10 test images, BA has better optimization ability than DE. Moreover, its variance of the fitness value is also the minimum in all algorithms. For “SHIP”, “SKIMAN”, “PERSON” and “BIRD” image, the variance is lower than $10^{-3}$ and the fitness value converges to the optimal solution, and other algorithms can not converge to the optimal solution for any test images, which illustrates that BA is a more stable and robust algorithm. In order to make a comparison between different chaotic strategies, Table 7 shows the average fitness value of 10 test images by using BA expect the local search and 11 different chaotic maps as the Section 3.2 showed. It is clear that the circle map is the best chaotic strategy among them; it has the maximum fitness value and minimum variance for all of the 10 test images. Although for “TARGET” and “GUN” images, the fitness value of tent map and gauss map is also very similar. For other seven test images, however, their fitness values are not very good. Moreover, Fan and Zhang [58] structured a new definition of piecewise map to carry out the chaotic process. In this paper, we proposed a novel piecewise circle map. The mapping curve and autocorrelation curve of the basic circle map and the piecewise circle map are respectively shown in Figure 2 and Figure 3, and the smaller autocorrelation coefficient is better [59]. Obviously, both the piecewise circle map and the basic circle map have a very small autocorrelation coefficient; they are as good enough as chaotic sequences. However, in contrast, the three maximum autocorrelation coefficients of the piecewise circle map and the basic circle map are 0.0713, 0.0694, 0.0632 and 0.0967, 0.0843, 0.0747, and the minimum are −0.1049, −0.0738, −0.0710 and −0.0986, −0.0835, −0.0757; respectively. Both the minimum and maximum autocorrelation coefficient of the piecewise circle map are all smaller than that of the basic circle map, and the average and variance of the piecewise circle map and the basic circle map are $-9.0106\times 10^{-20}$, $9.7632\times 10^{-4}$ and $6.81\times 10^{-4}$, 0.0010, while the average and variance of piecewise circle map are all much closer to 0. Hence, the piecewise circle map is more random and decentralized. The fitness value based on the piecewise circle map is better than that from using the basic circle map. The piecewise circle map is defined as Equation (31):

(31) $$x_{n+1}=\left\{\begin{array}{cc} \left(2x_{n}+\alpha -\left(\frac{\beta }{2\pi }\right)\sin\left(4\pi x_{n}\right)\right)\mathrm{mod}\;1, & x_{n}<0.5,\\ \left(1-\left(2\left(x_{n}-1\right)+\alpha -\left(\frac{\beta }{2\pi }\right)\sin\left(4\pi \left(x_{n}-1\right)\right)\right)\right)\mathrm{mod}\;1, & x_{n}\geq 0.5, \end{array}\right.$$

where $x_{n}$ denotes the value of the chaotic variable *x* at the nth iteration, x∈(0,1), while α and β are the control parameters; here, α=0.5, and β=0.2.

In addition, results of the proposed method are compared with three other methods, namely (i) local search before chaos using Equation (14) (ii) local search after chaos using Equation (14) and (iii) local search before chaos using Equation (28), which is shown in Table 8. It is obvious that Levy flight is a better-performing local search method. For all test images, its fitness value and variance are all better than that of Equation (14). On the other side, local search after chaos is a more reasonable idea; the chaotic process let the population have an extensive search space, and then the local search makes each individual around the optimal solutions. Finally, Table 9 shows the selected threshold and absolute error ratio of 10 test images. It is obviously revealed that GA, PSO, ACO, DE and basic BA can all make an optimization for 2D Tsallis entropy; the result is acceptable, but its threshold still has some differences compared with manual thresholding. The average threshold difference between those is more than 5; the maximum threshold difference is more than 10. However, the average threshold difference between the proposed method and manual thresholding is about 1, and the maximum threshold difference is only 5. At the same time, the absolute error ratio of the proposed method is lower than 0.2% for all of the 10 test images, and there are four test images for which absolute error ratio is as low as 0. The selected threshold of the proposed method is very close to manual thresholding, and its performance is better than other methods. The experiment shows that the proposed method has a better optimizing efficiency and convergence ability. The performance of the proposed method is better than other algorithms, which is more suitable for real-time image segmentation application using 2D Tsallis entropy.

### 5.2. Visual Evaluation of the Segmented Image

**Figure 4:**
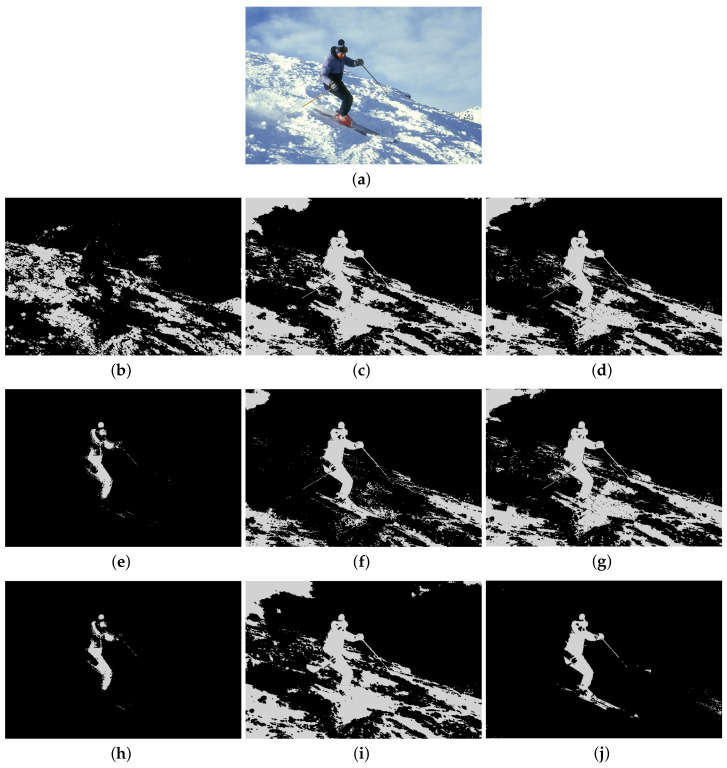
Segmented results of SKIMAN image: (**a**) original image; (**b**) 1D Fisher; (**c**) 1D cross entropy; (**d**) 1D maximum entropy; (**e**) 1D Tsallis entropy; (**f**) fuzzy entropy; (**g**) 2D Fisher; (**h**) 2D cross entropy; (**i**) 2D maximum entropy; (**j**) proposed method.

**Figure 5:**
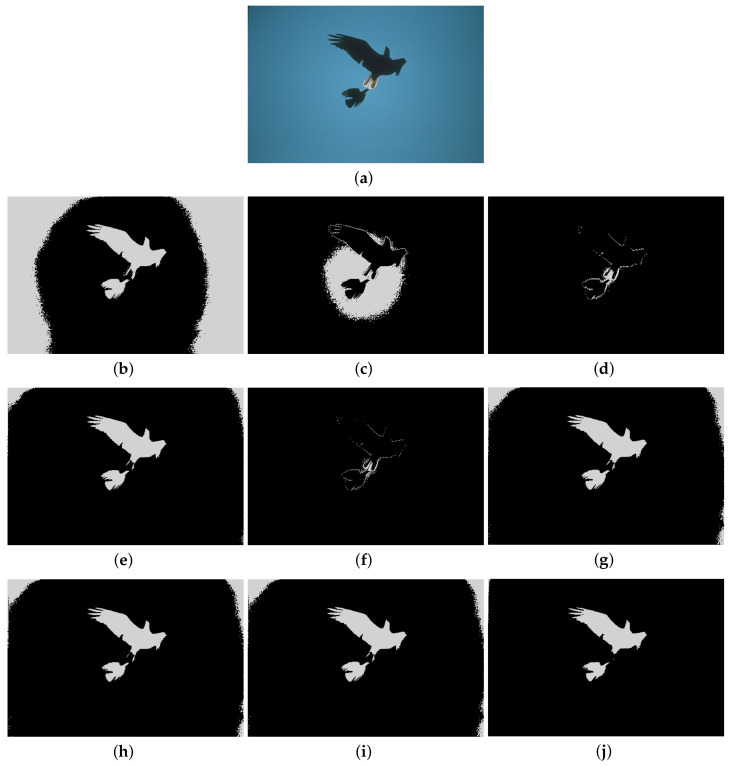
Segmented results of BIRD image: (**a**) original image; (**b**) 1D Fisher; (**c**) 1D cross entropy; (**d**) 1D maximum entropy; (**e**) 1D Tsallis entropy; (**f**) fuzzy entropy; (**g**) 2D Fisher; (**h**) 2D cross entropy; (**i**) 2D maximum entropy; (**j**) proposed method.

**Figure 6:**
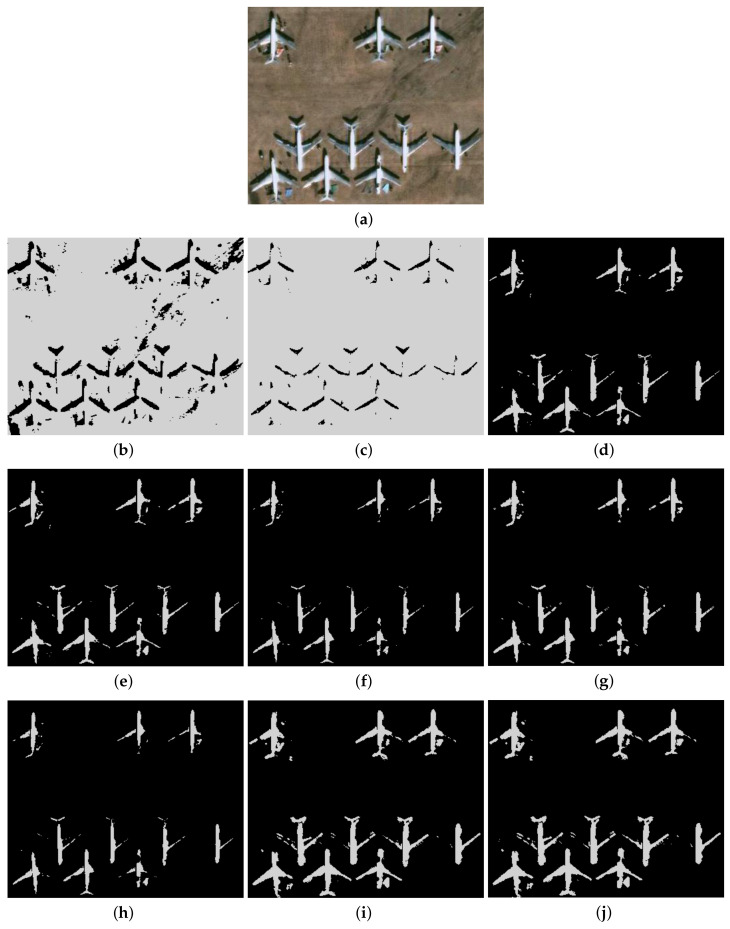
Segmented results of AIRPORT image: (**a**) original image; (**b**) 1D Fisher; (**c**) 1D cross entropy; (**d**) 1D maximum entropy; (**e**) 1D Tsallis entropy; (**f**) fuzzy entropy; (**g**) 2D Fisher; (**h**) 2D cross entropy; (**i**) 2D maximum entropy; (**j**) proposed method.

**Figure 7:**
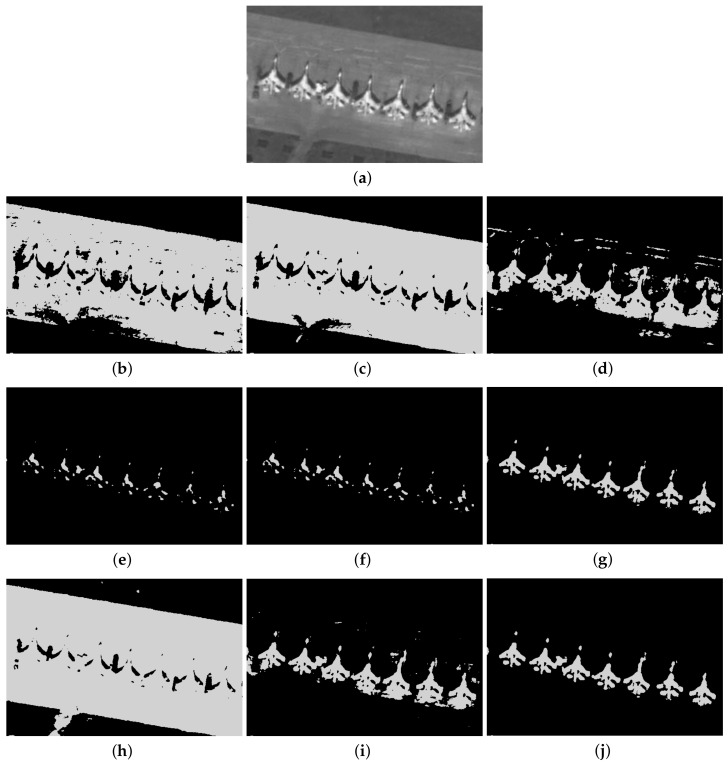
Segmented results of PLANE image: (**a**) original image; (**b**) 1D Fisher; (**c**) 1D cross entropy; (**d**) 1D maximum entropy; (**e**) 1D Tsallis entropy; (**f**) fuzzy entropy; (**g**) 2D Fisher; (**h**) 2D cross entropy; (**i**) 2D maximum entropy; (**j**) proposed method.

**Figure 8:**
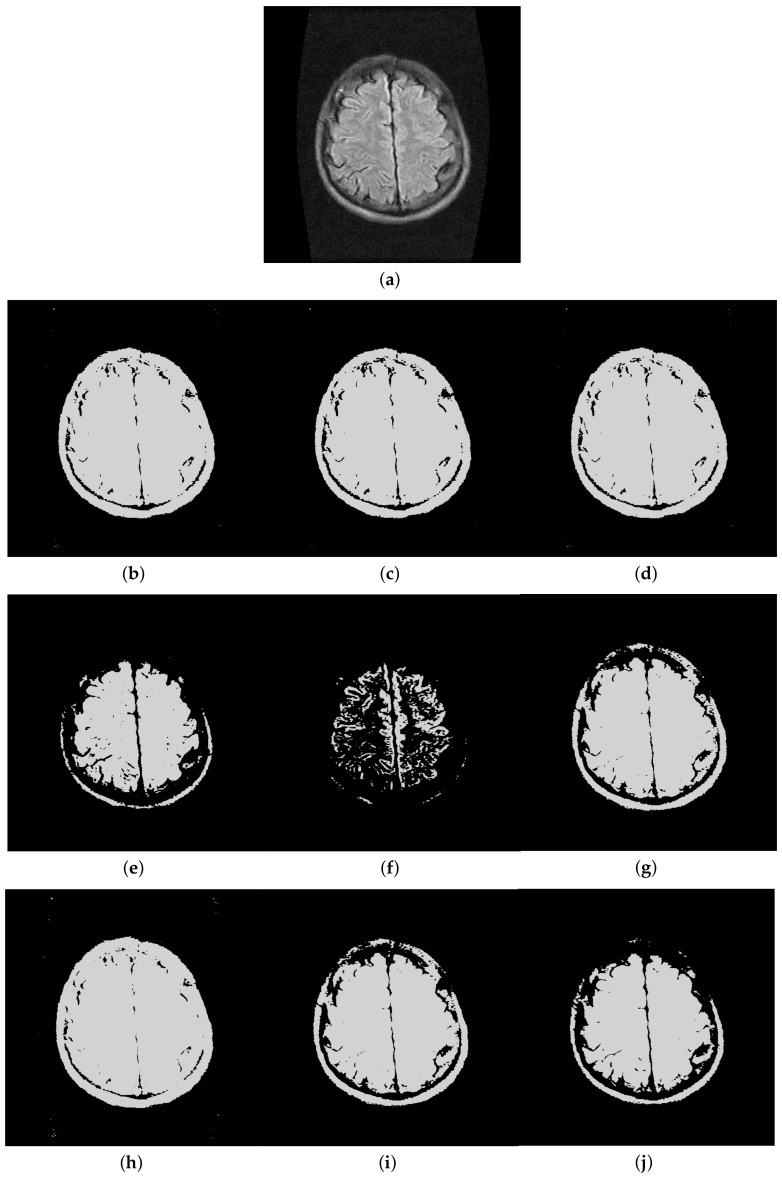
Segmented results of Magnetic Resonance Imaging (MRI) image: (**a**) original image; (**b**) 1D Fisher; (**c**) 1D cross entropy; (**d**) 1D maximum entropy; (**e**) 1D Tsallis entropy; (**f**) fuzzy entropy; (**g**) 2D Fisher; (**h**) 2D cross entropy; (**i**) 2D maximum entropy; (**j**) proposed method.

**Figure 9:**
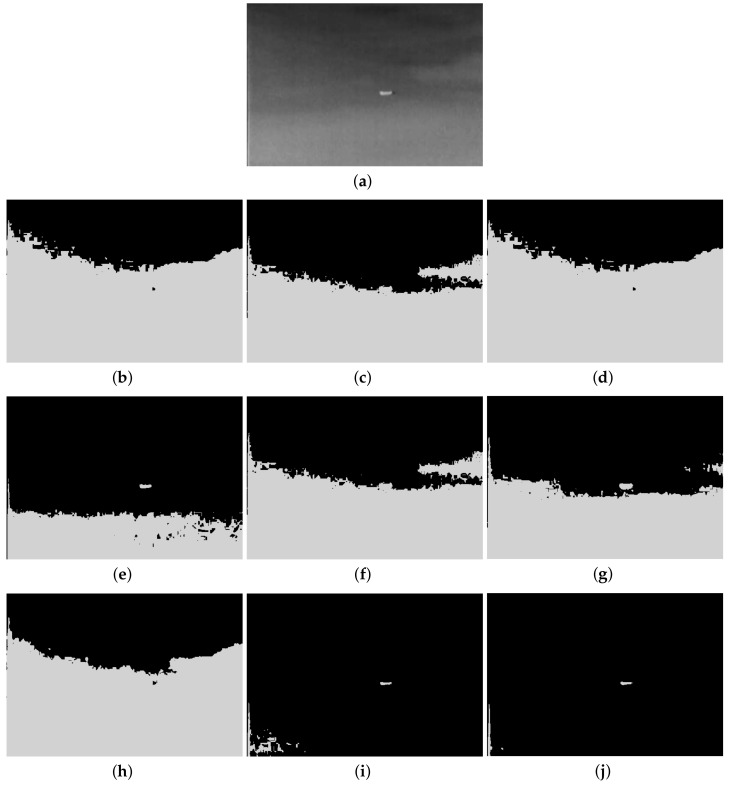
Segmented results of TARGET image: (**a**) original image; (**b**) 1D Fisher; (**c**) 1D cross entropy; (**d**) 1D maximum entropy; (**e**) 1D Tsallis entropy; (**f**) fuzzy entropy; (**g**) 2D Fisher; (**h**) 2D cross entropy; (**i**) 2D maximum entropy; (**j**) proposed method.

**Figure 10:**
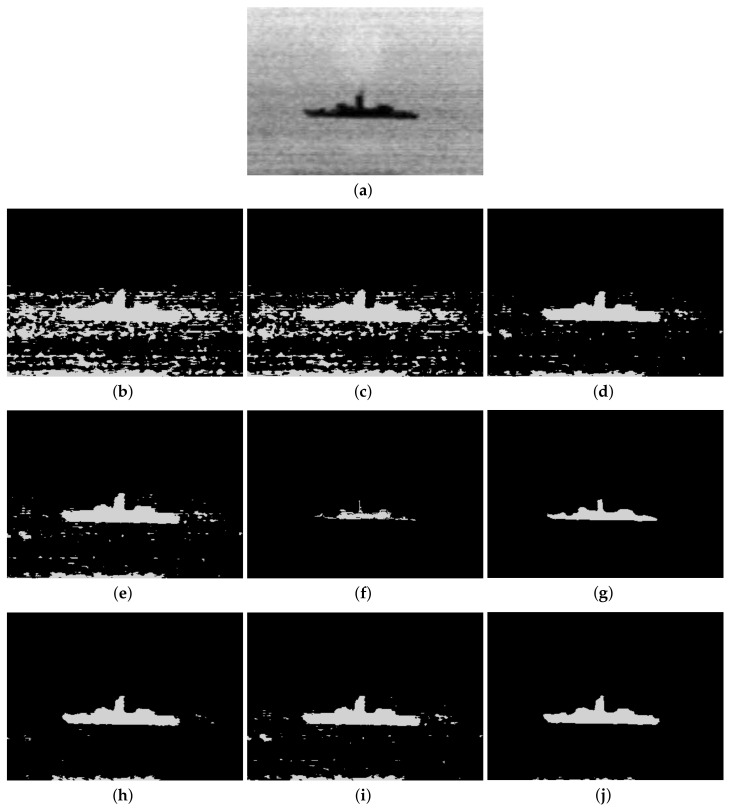
Segmented results of SHIP image: (**a**) original image; (**b**) 1D Fisher; (**c**) 1D cross entropy; (**d**) 1D maximum entropy; (**e**) 1D Tsallis entropy; (**f**) fuzzy entropy; (**g**) 2D Fisher; (**h**) 2D cross entropy; (**i**) 2D maximum entropy; (**j**) proposed method.

**Figure 11:**
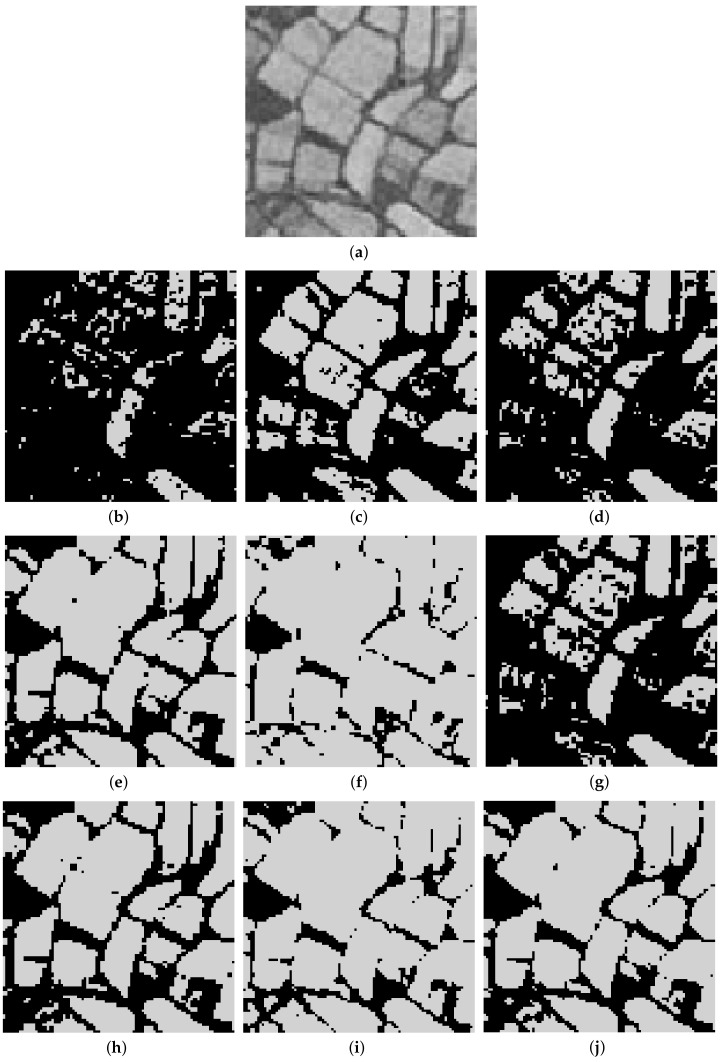
Segmented results of FIELD image: (**a**) original image; (**b**) 1D Fisher; (**c**) 1D cross entropy; (**d**) 1D maximum entropy; (**e**) 1D Tsallis entropy; (**f**) fuzzy entropy; (**g**) 2D Fisher; (**h**) 2D cross entropy; (**i**) 2D maximum entropy; (**j**) proposed method.

**Figure 12:**
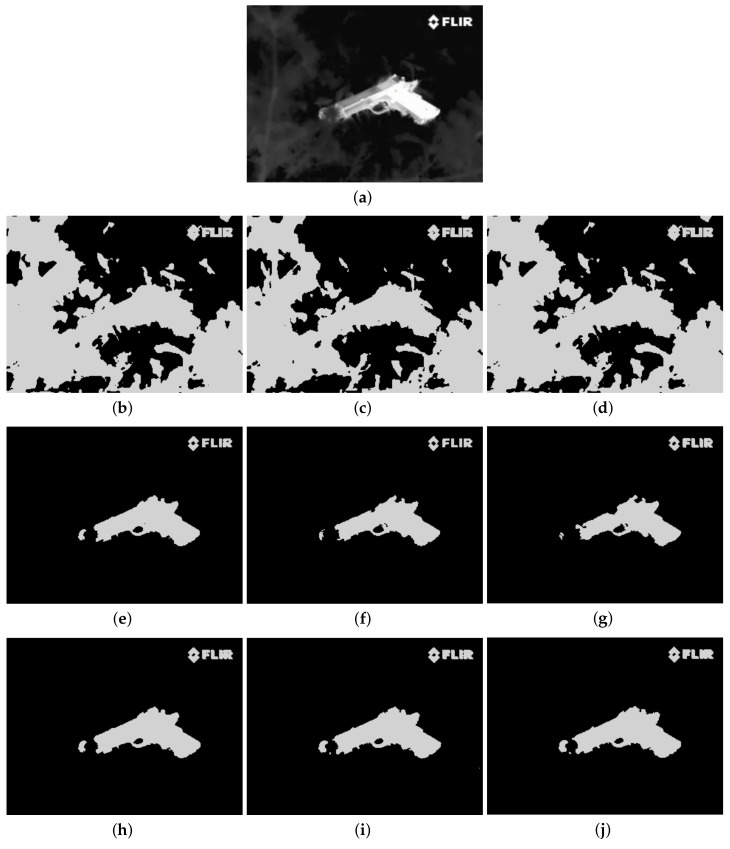
Segmented results of PISTOL image: (**a**) original image; (**b**) 1D Fisher; (**c**) 1D cross entropy; (**d**) 1D maximum entropy; (**e**) 1D Tsallis entropy; (**f**) fuzzy entropy; (**g**) 2D Fisher; (**h**) 2D cross entropy; (**i**) 2D maximum entropy; (**j**) proposed method.

**Figure 13:**
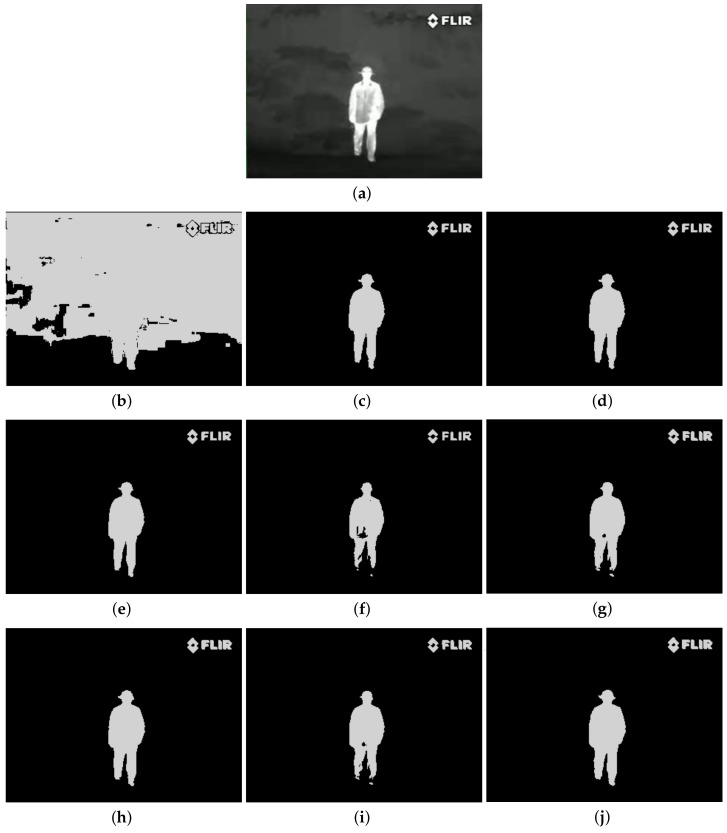
Segmented results of PERSON image: (**a**) original image; (**b**) 1D Fisher; (**c**) 1D cross entropy; (**d**) 1D maximum entropy; (**e**) 1D Tsallis entropy; (**f**) fuzzy entropy; (**g**) 2D Fisher; (**h**) 2D cross entropy; (**i**) 2D maximum entropy; **(j)** proposed method.

The qualitative performance of the proposed method and the contemporary methods are given in Figure 4, Figure 5, Figure 6, Figure 7, Figure 8, Figure 9, Figure 10, Figure 11, Figure 12 and Figure 13, respectively. The original images are shown in Figure 4a, Figure 5a, Figure 6a, Figure 7a, Figure 8a, Figure 9a, Figure 10a, Figure 11a, Figure 12a and Figure 13a. The segmented images of the same by 1D Fisher, 1D maximum entropy, 1D cross entropy, 1D Tsallis entropy, fuzzy entropy, 2D Fisher, 2D maximum entropy, 2D cross entropy and the proposed method are shown in Figure 4b–j, Figure 5b–j, Figure 6b–j, Figure 7b–j, Figure 8b–j, Figure 9b–j, Figure 10b–j, Figure 11b–j, Figure 12b–j and Figure 13b–j, respectively. According to the segmented images, 1D Tsallis entropy method has the best segmentation effect among all of the 1D histogram based thresholding methods. For some test images, the segmented result is even better than a portion of 2D histogram based thresholding method. Basically, all of the 2D histogram based thresholding methods have the better segmentation effect than corresponding 1D histogram based thresholding methods. Although 2D maximum entropy can make a great effect for four test images, for all of the 10 test images, its effect is not very good. The experiment shows that the MCBA based 2D Tsallis entropy algorithm provides the best thresholding performance among all methods being compared.

## 6. Conclusions

Automatically and adaptively selecting a robust, optimum threshold to separate an object from the background has been a hot topic in the field of image analysis. Among entropy-based methods, the methods based on Tsallis entropy has been proved to be an effective thresholding strategy and widely applied in image segmentation. However, entropy-based methods based on 1D histograms do not contain the spatial correlation information within the neighborhood. When the proportion of the target area is very small, its threshold of test image is very easy to drift or shift. Hence, some entropy methods based on 2D histograms containing the 2D Tsallis entropy method are proposed. To measure the segmentation capabilities, results of the proposed method are respectively compared with 1D Fisher, 1D cross entropy, 1D maximum entropy, 1D Tsallis entropy, fuzzy entropy, 2D Fisher, 2D maximum entropy and 2D cross entropy. Due to the 2D Tsallis entropy-based thresholding method having high computational costs, which requires a great deal of computation to seek a group of parameters, some meta-heuristics algorithms were employed to speed up the basic 2D Tsallis entropy-based thresholding method. However, these methods easily fall into the local optimum. Compared with chaotic maps of tent, iterative, gauss, dyadic, circle, cheby, sinus, singer, sinusoidal, sin, and logical, we proposed a novel piecewise circle map and proved its performance. In order to upgrade the search mechanism of the standard BA, chaotic sequences generated by the piecewise circle map and Levy flight are incorporated into the BA.

In the paper, MCBA is proposed and applied to the 2D Tsallis entropy algorithm for gray-level images segmentation, which employs MCBA to look for the best combination of all the parameters. Results of the proposed method are compared with some other meta-heuristics algorithms, like GA, ACO, DE, PSO, BA and CBA. Among these methods, the proposed method can converge to the optimal solution, and the segmentation results are better than other methods involved in this paper, which is a practical thresholding approach. However, the *q* parameter is not investigated here, which plays a crucial part as a tuning parameter in the image segmentation and is set a constant value. In future work, we will concentrate on (1) analysis of segmented results with different *q* values; and the (2) automatic and fine *q*-adjusting method.

## Figures and Tables

**Table 1:** Parameters used in the Bat algorithm.

Parameter	Explanation	Value
*N*	Number of bat(s)	30
Max_gen	Maximum iteration number	50
*L*	Gray-scale of test image	256
$r_{i}$	Rate of pulse emission	(0,1)
$A_{i}$	Loudness	(1,2)

**Table 2:** Parameters used in genetic algorithm (GA).

Parameter	Explanation	Value
*N*	Number of genetic(s)	30
Max_gen	Maximum iteration number	50
*L*	Gray-scale of test image	256
$P_{s}$	Selection ratio	0.9
$P_{c}$	Crossover ratio	0.8
$P_{m}$	Mutation ratio	0.05

**Table 3:** Parameters used in particle swarm optimization (PSO).

Parameter	Explanation	Value
*N*	Number of particle(s)	30
Max_gen	Maximum iteration number	50
*L*	Gray-scale of test image	256
$c_{1},c_{2}$	Positive acceleration constants	(0,2)
$r_{1},r_{2}$	Random numbers	(0,1)

**Table 4:** Parameters used in ant colony optimization algorithm (ACO).

Parameter	Explanation	Value
*N*	Number of ant(s)	30
Max_gen	Maximum iteration number	50
*L*	Gray-scale of test image	256
α,β	Relative importance of trail versus	1
ρ	Evaporation of pheromone	(0,1)

**Table 5:** Parameters used in differential evolution algorithm (DE).

Parameter	Explanation	Value
*N*	Number of individual(s)	30
Max_gen	Maximum iteration number	50
*L*	Gray-scale of test image	256
$f_{m}$	Mutation factor	0.54
CR	Crossover rate	0.6

**Table 6:** Land use classes in the Bagmati watershed at Khokana gauging station.

Image	Fitness Value/Variance									
	GA		PSO		ACO		DE		BA	
	Fitness	Variance	Fitness	Variance	Fitness	Variance	Fitness	Variance	Fitness	Variance
AIRPORT	83.1028	0.9902	83.3528	0.2585	83.4129	0.2714	83.4525	0.2391	83.5152	0.2052
PLANE	53.1643	0.5466	53.2977	0.3504	53.3612	0.2421	53.4279	0.1785	53.4454	0.1661
BIRD	32.6768	1.0563	34.6139	0.4007	35.1675	0.277	36.4929	0.1764	35.6323	0.137
SKIMAN	104.4435	0.1831	104.4531	0.1468	104.5013	0.0665	104.5476	0.0537	104.6098	0.046
MRI	56.0164	0.1244	56.0269	0.0935	56.0414	0.0794	56.0506	0.0786	56.0572	0.07
SHIP	75.4637	0.1754	75.4967	0.1568	75.5389	0.1265	75.5552	0.1156	75.5782	0.0976
FIELD	78.355	0.1862	78.3741	0.1748	78.4185	0.1571	78.4359	0.148	78.4559	0.1359
TARGET	29.6825	0.7195	29.8642	0.6341	29.9987	0.5543	30.1589	0.5195	30.2151	0.4113
PISTOL	49.9564	0.1145	49.9624	0.0885	49.9955	0.068	50.0008	0.0675	50.0104	0.0607
PERSON	54.0327	0.0795	54.0426	0.075	54.0571	0.0675	54.0666	0.0642	54.0794	0.0596

**Table 7:** Fitness value and variance of 10 test images using different chaotic maps.

**Image**	**Fitness Value/Variance**											
	**Logical**		**Sin**		**Sinusoidal**		**Singer**		**Sinus**		**Cheby**	
	**Fv.**	**Var.**	**Fv.**	**Var.**	**Fv.**	**Var.**	**Fv.**	**Var.**	**Fv.**	**Var.**	**Fv.**	**Var.**
AIRPORT	83.4556	0.1874	83.4476	0.2245	83.4069	0.2542	83.4477	0.2167	83.4015	0.2236	83.4016	0.2381
PLANE	53.4604	0.1499	53.4465	0.1782	53.4032	0.2073	53.4379	0.1871	53.3114	0.2812	53.408	0.2014
BIRD	35.2108	0.2488	35.2628	0.2211	35.2907	0.1925	34.7253	0.3986	35.4217	0.1635	35.3386	0.1813
SKIMAN	104.5371	0.1287	104.5166	0.1422	104.5127	0.1389	104.4798	0.1749	104.514	0.1445	104.5233	0.1328
MRI	56.0493	0.0508	56.0375	0.0599	56.06	0.046	56.0333	0.0616	56.0598	0.0361	56.0569	0.0378
SHIP	75.5802	0.0993	75.5699	0.0831	75.5589	0.0911	75.5465	0.1175	75.569	0.0943	75.566	0.0779
FIELD	78.4559	0.1365	78.4335	0.1441	78.3985	0.161	78.4069	0.1558	78.4072	0.1511	78.3902	0.1664
TARGET	30.0874	0.4777	30.1089	0.451	30.3689	0.4687	30.0806	0.4875	30.0907	0.4633	30.0189	0.5151
PISTOL	49.9834	0.07	49.9777	0.0798	49.9807	0.063	49.9715	0.0813	50.0054	0.0619	49.9863	0.0693
PERSON	54.0503	0.0652	54.0716	0.0623	54.0661	0.0645	54.0462	0.0692	54.0746	0.0551	54.0678	0.0549
**Image**	**Circle**		**Dyadic**		**Gauss**		**Iterative**		**Tent**		**Proposed**	
	**Fv.**	**Var.**	**Fv.**	**Var.**	**Fv.**	**Var.**	**Fv.**	**Var.**	**Fv.**	**Var.**	**Fv.**	**Var.**
AIRPORT	83.5375	0.1639	83.4889	0.1701	83.4244	0.2483	83.3773	0.2395	83.5044	0.171	83.5573	0.1489
PLANE	53.4858	0.1391	53.4493	0.1672	53.3666	0.2266	53.3249	0.2756	53.4277	0.2045	53.4949	0.1197
BIRD	35.8136	0.1217	35.4958	0.1442	35.5737	0.1487	35.569	0.1434	35.484	0.1583	35.8509	0.1171
SKIMAN	104.5839	0.0805	104.5375	0.1224	104.5459	0.1118	104.5556	0.1081	104.5556	0.1128	104.6035	0.0612
MRI	56.0704	0.0336	56.0653	0.0411	56.054	0.0499	56.0624	0.0408	56.0698	0.0391	56.0855	0.0306
SHIP	75.6066	0.0586	75.5978	0.0736	75.5693	0.0855	75.5283	0.1227	75.5802	0.0708	75.6082	0.0582
FIELD	78.4766	0.1184	78.4129	0.1508	78.4192	0.1465	78.3819	0.1771	78.4446	0.1435	78.4855	0.1139
TARGET	30.2985	0.3826	30.121	0.4404	29.9963	0.5143	30.0074	0.4968	30.2087	0.4175	30.4515	0.3486
PISTOL	50.0119	0.0556	49.9997	0.0643	50.0003	0.0644	50.0001	0.0596	49.9998	0.0669	50.0176	0.055
PERSON	54.0898	0.0506	54.0753	0.0625	54.0864	0.0538	54.0839	0.0561	54.0571	0.0546	54.0956	0.0469

**Table 8:** Fitness value and variance of 10 test images using different methods.

Image	Fitness Value/Variance							
	(i)		(ii)		(iii)		Proposed	
	Fitness	Variance	Fitness	Variance	Fitness	Variance	Fitness	Variance
AIRPORT	83.5953	0.114	83.626	0.0799	83.674	0.059	83.694	0.039
PLANE	53.5156	0.0946	53.594	0.0749	53.6349	0.0357	53.6479	0.0061
BIRD	36.1578	0.0721	36.1643	0.0427	36.1937	0.0075	36.1979	$5.15\times 10^{-6}$
SKIMAN	104.6216	0.0451	104.6375	0.0284	104.6554	0.006	104.6579	$6.61\times 10^{-5}$
MRI	56.0961	0.0188	56.0976	0.0179	56.1109	0.015	56.1206	0.0074
SHIP	75.6485	0.0216	75.6496	0.0209	75.6651	0.0069	75.6691	$8.61\times 10^{-8}$
FIELD	78.5885	0.0919	78.6006	0.0833	78.6465	0.0706	78.6612	0.0523
TARGET	30.5192	0.2856	30.5275	0.2787	30.804	0.1424	30.9007	0.1261
PISTOL	50.0588	0.0372	50.0734	0.0193	50.0789	0.0119	50.0847	0.0041
PERSON	54.1278	0.0255	54.1351	0.0233	54.1471	0.0084	54.1501	$9.53\times 10^{-4}$

**Table 9:** Selected threshold and error ratio (${\%}_{o}$) of 10 test images.

Image	Size	Ref. Value	Selected Threshold/Error Ratio (${\%}_{o}$)											
			GA		PSO		ACO		DE		BA		MCBA	
			Threshold	Error	Threshold	Error	Threshold	Error	Threshold	Error	Threshold	Error	Threshold	Error
AIRPORT	355 × 297	145,144	156,151	10.1	159,146	8.8	151,149	6.7	148,148	4.7	147,146	2.7	145,146	1.6
PLANE	540 × 360	157,157	146,158	13	154,152	5.9	161,160	3.8	159,161	3.4	162,157	2.1	158,159	1.7
BIRD	481 × 321	8973	110,93	26.4	9883	11.6	8983	7.1	9279	3.3	8871	1.9	8973	0
SKIMAN	481 × 321	110,112	11,195	3.4	128,110	2.1	105,110	1.9	106,115	1.7	113,112	1	110,112	0
MRI	512 × 512	106,106	119,111	10	11,099	8.5	102,101	6.8	100,105	5.9	109,108	3.2	106,105	0.97
SHIP	360 × 256	105,127	121,126	8.4	117,128	6.1	118,123	5.4	115,125	4.3	112,128	3.3	105,127	0
FIELD	100 × 100	131,132	144,137	47.4	140,136	35.3	136,137	28.2	137,130	13.7	128,129	8	132,132	1.8
TARGET	552 × 381	150,150	158,143	11.6	145,162	5.5	152,162	3.4	153,161	1.1	157,151	0.86	153,152	0.39
PISTOL	320 × 240	7575	7469	1.7	8678	1.6	7981	1.2	7571	0.77	7177	0.56	7573	0.14
PERSON	320 × 236	104,112	121,111	1.4	111,118	0.93	105,108	0.73	109,113	0.52	101,111	0.19	104,112	0

## Supplementary Materials

Supplementary material is available online at http://www.mdpi.com/1099-4300/20/4/239/s1.
